# Gender-affirming medical treatment for adolescents: a critical reflection on “effective” treatment outcomes

**DOI:** 10.1186/s12910-024-01143-8

**Published:** 2024-12-24

**Authors:** Ezra D. Oosthoek, Skye Stanwich, Karl Gerritse, David Matthew Doyle, Annelou L.C. de Vries

**Affiliations:** 1 Center of Expertise on Gender Dysphoria (CEGD), Amsterdam UMC, Vrije Universiteit, De Boelelaan 1118, Amsterdam, 1081 HZ The Netherlands; 2 Department of Child and Adolescent Psychiatry, Amsterdam UMC, Vrije Universiteit, De Boelelaan 1118, Amsterdam, 1081 HZ The Netherlands; 3 Department of Medical Psychology, Amsterdam UMC, Vrije Universiteit, De Boelelaan 1118, Amsterdam, 1081 HZ The Netherlands

**Keywords:** Gender-affirming medical treatment, Trans and gender diverse adolescents, Effective treatment outcomes, Trans negativity, Improvement

## Abstract

**Background:**

The scrutiny surrounding gender-affirming medical treatment (GAMT) for youth has increased, particularly concerning the limited evidence on long-term treatment outcomes. The Standards of Care 8 by the World Professional Association for Transgender Health addresses this by outlining research evidence suggesting “effective” outcomes of GAMT for adolescents. However, claims concerning what are considered “effective” outcomes of GAMT for adolescents remain implicit, requiring further reflection.

**Methods:**

Using trans negativity as a theoretical lens, we conducted a theory-informed reflexive thematic analysis of the literature cited in the “Research Evidence” section of the SOC8 Adolescents chapter. We selected 16 articles that used quantitative measures to assess GAMT outcomes for youth, examining how “effective” outcomes were framed and interpreted to uncover implicit and explicit normative assumptions within the evidence base.

**Results:**

A total of 44 different measures were used to assess GAMT outcomes for youth, covering physical, psychological, and psychosocial constructs. We identified four main themes regarding the normative assumptions of “effective” treatment outcomes: (1) *doing bad: experiencing distress before GAMT*, (2) *moving toward a static gender identity and binary presentation*, (3) *doing better: overall improvement after GAMT*, and (4) the *absence of regret*. These themes reveal implicit norms about what GAMT for youth should achieve, with improvement being the benchmark for “effectiveness.”

**Discussion:**

We critically reflect on these themes through the lens of trans negativity to challenge what constitutes “effective” GAMT outcomes for youth. We explore how improvement justifies GAMT for youth and address the limitations of this notion.

**Conclusions:**

We emphasize the need for an explicit discussion on the objectives of GAMT for adolescents. The linear narrative of improvement in GAMT for adolescents is limited and fails to capture the complexity of GAMT experiences. With currently no consensus on how the “effectiveness” of GAMT for adolescents is assessed, this article calls for participatory action research that centers the voices of young TGD individuals.

## Introduction

This article appears during a critical moment in the provision of gender-affirming medical treatment^1^ (GAMT) for youth. Discussions around the safety and efficacy of GAMT for youth are becoming increasingly widespread, along with growing criticism regarding the lack of longer-term evidence for GAMT outcomes. Given this context, we believe it is relevant to reflect on what evidence substantiates “effective” outcomes of GAMT for adolescents, and to examine which treatment outcomes justify and legitimize GAMT for adolescents. Thereby, we seek to open up conversations about what *good* research evidence for GAMT for youth consists of. To do so, this article reviews the cited literature in the “Research evidence of gender-affirming medical treatment for transgender adolescents” section of the Standards of Care 8 (SOC8), developed by the World Professional Association for Transgender Health (WPATH) (1 pp45-47). This section addresses the evidence base of GAMT for adolescents by outlining research evidence that presents “effective” outcomes. However, claims concerning what should be considered “effective” outcomes of GAMT for adolescents remain implicit within the document, leaving room for further reflection.

Drawing from the interdisciplinary field of *trans studies*, we introduce trans negativity as a theoretical lens to critically reflect on the implicit and explicit normative assumptions underlying “effective” GAMT outcomes. Trans negativity examines how negative affect – such as distress and suffering – plays a crucial role in both medical and socio-cultural narratives surrounding gender transition. It highlights how negative affect is deeply embedded in the pathologization and medicalization of TGD identities and the curative discourse surrounding GAMT, and critiques the framing of negative affect as a problem to be resolved through medical intervention. Using this perspective, we critically analyze the normative assumptions underpinning the SOC8 research evidence and argue for acknowledging the intrinsic complexity of GAMT, including the often enduring presence of negative affect throughout and beyond transition [1–3]. In doing so, this paper ultimately seeks to reflect on GAMT treatment outcomes used as benchmarks for “effectiveness” and to foster discussion on the ethical question of what constitutes *good* outcomes of GAMT for adolescents.

While the existence of trans and gender-diverse^2^ (TGD) youth seeking gender-affirming medical care is not a new phenomenon [4], GAMT for youth has developed rapidly in the past two decades, particularly concerning puberty suppression and hormone replacement therapy [5, 6]. As the visibility of (young) TGD individuals has increased, so too has the scrutiny surrounding GAMT for this demographic, prompting increasingly polarizing debates within both the healthcare community and society at large [7–11]. The controversies surrounding GAMT for youth are reflected in the growing number of restrictions on the provision of this care in various countries [12]. In the United States, an increasing number of anti-trans bills are restricting – and even criminalizing – this care practice [13, 14]. These limitations to accessing GAMT are increasingly paralleled in Europe. For example, in Sweden, pediatric endocrinologists now only prescribe puberty suppression under strict conditions [15]. Similarly, NHS England recently made the decision to reorganize the provision of GAMT for TGD youth and restrict the prescription of puberty suppression, further highlighting the precarity of this medical treatment [16–18].

A central aspect of the scrutiny leveraged at this care practice is the critique that there is insufficient evidence regarding the treatment outcomes of GAMT for youth, which contributes to rising uncertainties about the long-term effects of treatment [19, 20]. In turn, some concerned critics have deemed the current research to be insufficient and have called for a strengthening of the evidence base for GAMT for youth [18, 21, 22]. Not only are questions arising regarding the *quantity* of research in this field, but critics are also concerned with the *quality* of the available evidence [21]. Recent systematic reviews have graded the quality of the current evidence base for this care as relatively low, due primarily to the absence of randomized controlled trials [23, 24]. Understandably, clinicians and clients alike seek to base treatment decisions on the best available evidence; a high quantity and quality of evidence enables healthcare providers to feel reassured in medical decision-making and helps to provide a sense of order in navigating the uncertainty that is inherent to medical care in general, and GAMT for youth in particular [25, 26]. This heightened uncertainty around GAMT for adolescents relates to concerns about their capacity to make long-term decisions about their future [22, 27, 28], especially when it comes to decisions about fertility and the potential risk of regret later in life [19, 29, 30]. These concerns are intertwined with the understanding of adolescence as a critical developmental period during which identity is still in development. Some argue that no GAMT interventions – especially irreversible ones – should be performed during this stage [19, 21].

Furthermore, typical evidence-based medicine practices (i.e., relying on scientific evidence and clinical expertise) may not always help to inform decision-making in this context [25]. Meeting the “gold-standard” of evidence-based medicine can help to legitimize the provision of care, protecting against criticism that treatment approaches are unfounded [31–33]. However, some scholars argue that meeting this “gold-standard” through performing randomized controlled trials in transgender adolescent care is both methodologically inappropriate and unethical as it may deny or delay treatment, thereby making it difficult to recruit participants willing to risk being assigned to a non-treatment group [27, 34]. Given this context, the “gold-standard” of evidence-based medicine has not been considered suitable for guiding research practices for GAMT for youth, which is believed to contribute to uncertainty around the legitimacy of this care practice and its evidence base [25]. Despite the criticism surrounding GAMT for youth and its corresponding evidence base, experts within the field of transgender adolescent care have aimed to systemize care by integrating available evidence, patient values, and clinical expertise to create care models [26, 35].

### WPATH and the SOC8

Currently, the most widely adopted care model for GAMT is likely the SOC by the WPATH, an international, multidisciplinary, professional association whose mission is to promote evidence-based transgender healthcare [35]. The first version of the SOC, published over four decades ago when GAMT was not yet regulated as it currently is, sought to establish quality guidelines for this care practice. Furthermore, the SOC outlined eligibility criteria to determine who may qualify for GAMT interventions,^3^ such as hormone replacement therapy and/or surgical care [35, 36]. Since its inception, experts within WPATH have continuously collaborated on and updated the SOC, which are now widely implemented in local care guidelines in multidisciplinary clinics around the world. The latest version, the SOC8 (released in 2022),^4^ provides guidance on various aspects of trans-specific healthcare, including mental health, puberty suppression therapy, hormone replacement therapy, and surgical care.

In response to the growing demand for a more robust evidence base, the authors of the SOC8 state that the current document is “based upon a more rigorous and methodological evidence-based approach than previous versions,” building on published literature as well as consensus-based expert opinion (1 p8). The present state of the field consists of a limited number of GAMT *outcome studies* that follow TGD youth throughout their treatment trajectories into adulthood. As a result, the authors of the SOC8 Adolescents chapter point out that they could not conduct a systematic literature review of treatment outcomes in adolescents akin to that executed in, for instance, the chapter on Hormone Therapy [35]. Instead, a short narrative review on GAMT outcomes in adolescents was provided in the SOC8 Adolescents chapter, suggesting that the evidence base for GAMT for youth is in fact growing, and the best available evidence “indicates [that] providing gender-affirming treatment for gender diverse youth who meet criteria leads to *positive* outcomes” and “can be *effective* and helpful for many transgender adolescents” (1 p47,65, emphases added). However, the assertion of “effective” and “positive” outcomes of GAMT for adolescents raises questions about the underlying value judgments defining what constitutes desirable outcomes, which often remain implicit in the literature.

Given this background, this article aims to address the following questions: what are considered “effective” treatment outcomes in the SOC8 Adolescents chapter and how are normative assumptions regarding GAMT reflected through these outcomes? Informed by trans negativity as a theoretical lens, we perform a theory-driven reflexive thematic analysis of the cited literature in the “Research Evidence” section in the SOC8 Adolescents chapter. We analyze the cited literature in this particular section of the SOC8 Adolescents chapter with the aim to (1) provide an overview of the treatment outcomes in the cited literature of the research evidence in the SOC8 Adolescents chapter used to substantiate “effective” outcomes of GAMT for adolescents and (2) to elucidate normative assumptions underlying these treatment outcomes. By examining these assumptions through the lens of trans negativity, we seek to gain a more explicit understanding of how “effective” GAMT outcomes are defined throughout the evidence-base and interpreted by the SOC8 authors, as well as the underlying values that shape these definitions. To be clear, this article does not seek to question the importance or necessity of GAMT, but rather to critically reflect on claims regarding its “effectiveness” for TGD adolescents and how these claims are used to justify this care practice. Through our analysis, we aim to open up the conversation on what constitutes “effective” GAMT outcomes, particularly in relation to the diverse and often complex transition experiences of TGD youth.

### Theoretical lens: trans negativity

The prevailing narrative of being “born in the wrong body” has long dominated the medical discourse on TGD individuals, often portraying GAMT as a “cure” aimed at aligning individuals with dominant heteronormative gender norms [37–39]. TGD individuals have often been depicted as deviating from the norm, in turn framing medical gender transition as a means to attain a congruent, (hetero)normative gender identity and gender expression [37, 39–41]. Historically, the pathologization and medicalization of TGD experiences have been pervasive in both the medical community and society at large [42]. While the field of GAMT is making efforts to move away from the pathologization of TGD individuals and towards destigmatization and affirmation of TGD identities, the medical diagnoses of gender dysphoria [43] and gender incongruence [44] persist in order to validate and insure the provision of GAMT [35, 45, 46].

Critiquing the pathologization and medicalization of TGD individuals, *trans studies* was established as an interdisciplinary field to address the historical erasure and exclusion of trans and gender non-conforming people within academic discourse [47]. *Trans studies* is concerned with the study of gender and the experiences of TGD individuals, examining social, cultural, historical, political, and medical facets of gender while also exploring other intersections such as race, ethnicity, class, and disability. Central to *trans studies* are critiques of the normative biomedical framing of gender transition challenging restrictive binary narratives that depict gender transition as a linear journey from man to woman or vice versa [48, 49].

An important aspect of this critique, informed by feminist and queer affect theory, is the role of negative affect – or *feeling bad* – in the context of GAMT. Feminist and queer affect theory (e.g., [50–53]) offers tools to explore how affective experiences are shaped by cultural, social, and political forces. This framework provides a useful lens for understanding the persistence of negative feelings throughout and beyond gender transition, challenging the dominant view of GAMT as a linear, teleological process aimed at achieving alignment between one’s gender identity and body, ultimately leading to a coherent sense of self. This prevailing narrative is shaped by the expectation that transition should lead to *improvement*, implying that each step in the transition process mitigates negative feelings, ultimately “curing” gender dysphoria and improving the well-being of the TGD individual [2, 39]. Importantly, this perspective extends beyond the medical community; it is also prominent among TGD activists and advocates for transgender healthcare who emphasize the life-saving potential of gender-affirming interventions, particularly in preventing suicide and improving life satisfaction [54].

While theorizations on the persistence of negative affect throughout and beyond GAMT encourage critical reflection on the premise of GAMT as leading to physical alignment as well as psychological and psychosocial *improvement*, these insights have primarily remained within the realm of trans cultural theory, with limited integration into biomedical research or transgender healthcare [1–3]. As authors, we see value in bridging these disciplines to foster more nuanced and interdisciplinary conversations within transgender healthcare.

## Methods

We conducted a theory-informed reflexive thematic analysis of textual materials to examine the literature cited in the “Research Evidence” section in the SOC8 Adolescents chapter [55, 56]. Our dataset consisted of 16 articles^5^ from this section of the SOC8, which we selected based on their use of quantitative outcome measures to assess GAMT for youth. The articles were selected from a total of 24 cited sources in the “Research Evidence” section. For the purposes of our analysis, we chose to focus only on literature that presented treatment outcomes of GAMT. Sources that were excluded used qualitative methods [57, 58], extracted data from medical files or care records [59–61], were case presentations or reports [27, 62], or focused on detransition needs [63]. We recorded each of the outcome measures utilized in these sources and subsequently grouped these into six categories (see Results). These categorizations were developed based on how the authors in the cited sources used, described and employed each measure, reflecting the intended outcomes the measures aimed to assess. Importantly, many of these measures are broadly defined, overlapping, or are used by various authors in different ways.

We focused our thematic analysis on the introduction, discussion, and conclusion sections of these articles to gain comprehensive insights into the study aims and authors’ interpretations regarding “effective” GAMT outcomes. A specific focus on these sections allowed us to understand how the authors framed their studies and interpreted GAMT outcomes, highlighting what were considered “effective” outcomes of GAMT for adolescents. Although we did not code the remaining sections for the thematic analysis, we reviewed the methods and results sections of these articles to document the wide variety of measures used in each study (see Table 1). In line with the principles of reflexive thematic analysis, our approach was intentionally subjective [55, 56]; our codes and themes represent our interpretations, and these are informed by our subjectivities and the theoretical lens through which we approach this research. Throughout the analysis process, the author team frequently reflected upon and discussed our interpretations of the data with one another.

**Table 1:** Utilized scales to evaluate gender-affirming medical treatment outcomes for adolescents

Scale	Object of measure	Source
Body Image Scale (BIS)	Body image satisfaction or dissatisfaction	Carmichael et al. (2021) [20]
	Satisfaction With Primary, Secondary, and Other Physical Characteristics	Cohen-Kettenis & van Goozen (1997) [64]
	Body satisfaction	De Vries et al. (2011) [65]
	Body image	De Vries et al. (2014) [6]
	Body image dissatisfaction	Grannis et al. (2021) [66]
	Body dissatisfaction	Kuper et al. (2020); [67] Smith et al. (2001; 2005) [29, 68]
Utrecht Gender Dysphoria Scale (UGDS)	Intensity of gender dysphoria	Carmichael et al. (2021) [20]
	Gender dysphoria	Cohen-Kettenis & van Goozen (1997) [64]; Smith et al. (2001; 2005) [29, 68]; De Vries et al. (2011; 2014) [6, 65]
	GD-related discomfort	Costa et al. (2015) [69]
Youth Self Report (YSR)	Emotional and behavioral problems (psychological functioning)	Becker-Hebly et al. (2021) [19]
	General psychological functioning, self-harm	Carmichael et al. (2021) [20]
	Behavioral and emotional problems	De Vries et al. (2011; 2014) [6, 65]
	Internalizing and externalizing problem behavior, self-harm/suicidality, and poor peer relations	Van der Miesen et al. (2020) [70]
Children’s Global Assessment Scale (CGAS)	Global functioning	Becker-Hebly et al. (2021) [19]; De Vries et al. (2014) [6]
	Psychological and social functioning	Carmichael et al. (2021) [20]
	Global Psychosocial Functioning	Costa et al. (2015) [69]
	Overall severity of disturbance in children	De Vries et al. (2011) [65]
Minnesota Multiphasic Personality Inventory (MMPI) or Dutch version (NVM)	Personality inventory: negativism, somatization, shyness, psychopathology, and extroversion	Cohen-Kettenis & van Goozen (1997) [64]
	Psychological functioning	Smith et al. (2001; 2005) [29, 68]
Child Behaviour Checklist (CBCL)	General psychological functioning, self-harm	Carmichael et al. (2021) [20]
	Behavioral and emotional problems	De Vries et al. (2011; 2014) [6, 65]
Adult Self Report (ASR)	Emotional and behavioral problems (psychological functioning)	De Vries et al. (2014) [6] Becker-Hebly et al. (2021) [19]
Treatment evaluation questionnaire (self-constructed) - satisfaction with surgery	Functionality of the vagina or penis	Cohen-Kettenis & van Goozen (1997) [64]
	Functionality of the vagina or penis and satisfaction with surgical results	Smith et al. (2001) [29]
	Functioning of vagina/penis and breasts	Smith et al. (2005) [68]
Trait Anxiety Scale (STAI) of State-Trait Personality Inventory	Tendency to respond with anxiety to a threatening or annoying situation	De Vries et al. (2011; 2014) [6, 65]
Trait Anger Scale (TPI) of State-Trait Personality Inventory	Tendency to respond with anger to a threatening or annoying situation	De Vries et al. (2011; 2014) [6, 65]
Beck Depression Inventory (BDI)	Presence and degree of depression	De Vries et al. (2011) [65]
	Presence and severity of depressive symptoms	De Vries et al. (2014) [6]
Screen for Child Anxiety Related Emotional Disorders (SCARED)	Symptoms of generalized anxiety	Grannis et al. (2021) [66]
	Panic-related, social, separation-related, generalized, and school avoidance–related anxiety symptoms	Kuper et al. (2020) [67]
Appraisal of Appearance Inventory (AAI)	(In)compatibility of the appearance with the new gender	Smith et al. (2001) [29]
	Gender compatibility	Smith et al. (2005) [68]
Dutch version of Symptom Checklist 90 (SCL-90)	Psychological functioning/stability	Smith et al. (2001; 2005) [29, 68]
Patient Health Questionnaire (PHQ-9)	Symptoms of depression	Achille et al. (2020) [71]
	Depression	Tordoff et al. (2022) [72]
The Social Support Scale	Enquiry about the patient’s eight closest acquaintances	Smith et al. (2005) [68]
The Center for Epidemiological Studies Depression Scale (CESD-R)	Psychological measure - depression	Achille et al. (2020) [71]
Quality of Life Enjoyment and Satisfaction (QLES-Q-SF)	Psychological measure - quality of life	Achille et al. (2020) [71]
Ask Suicide-Screening (ASQ)	Suicidality	Allen et al. (2019) [73]
General Well-Being Scale (GWBS) of the Pediatric Quality of Life Inventory	Well-being	Allen et al. (2019) [73]
Kidscreen-27	Health related quality of life - psychological and physical dimensions	Becker-Hebly et al. (2021) [19]
Short Form-8	Mental and physical quality of life	Becker-Hebly et al. (2021) [19]
Dutch Personality Questionnaire (NPV)	Feelings of inadequacy, social inadequacy, rigidity, hostility, complacency, dominance, and self-esteem	Cohen-Kettenis & van Goozen (1997) [64]
Social Reactions Questionnaire	Reactions of the social environment to the transsexual	Cohen-Kettenis & van Goozen (1997) [64]
Adult Behavioral Checklist (ABCL)	Behavioral and emotional problems	De Vries et al. (2014) [6]
WHOQOL-BREF	Quality of life, subjective well-being	De Vries et al. (2014) [6]
The Satisfaction With Life Scale (SWLS)	Life satisfaction	De Vries et al. (2014) [6]
Subjective Happiness Scale (SHS)	Happiness	De Vries et al. (2014) [6]
Quick Inventory of Depressive Symptoms (QIDS)	Symptoms of depression	Kuper et al. (2020) [67]
Public Confrontation Questionnaire	Evaluate the subject’s experiences of being able to “pass” in the new social role	Smith et al. (2001) [29]
The Affect Balance Scale	Overall psychological well-being	Smith et al. (2001) [29]
GID in Childhood Scale	Presence of GID symptoms in childhood	Smith et al. (2005) [68]
Generalized Anxiety Disorder (GAD-7)	Anxiety, self-harm and suicidal thoughts	Tordoff et al. (2022) [72]
Connor-Davidson Resilience Scale (CD-RISC)	Resilience	Tordoff et al. (2022) [72]
LSAS	Social anxiety	Grannis et al. (2021) [66]
CDI	Depression	Grannis et al. (2021) [66]
SBQ-R	Suicidality/non-suicidal self-injury	Grannis et al. (2021) [66]
Client Satisfaction Questionnaire (ZUF-8)	Youth’s satisfaction with Transition related care (TRC)	Nieder et al. (2021) [74]
Individual Treatment Progress Score (ITPS)	Individual progress with regard to completion of a medical transition	Nieder et al. (2021) [74]
F-SozU	Adolescents’ perceived social support	Nieder et al. (2021) [74]
Kessler-6 Psychological Distress Scale	Severe psychological distress	Turban et al. (2022) [75]
Kidscreen-52	Health related quality of life	Carmichael et al. (2021) [20]
Treatment evaluation and post-treatment functioning	Post-operative functioning and (dis)satisfaction	Smith et al. (2005) [68]
Post-operative Functioning Scale	Postoperative functioning and satisfaction with SR	Smith et al. (2005) [68]
Life circumstances (self-constructed)	Objective and Subjective Well-Being	De Vries et al. (2014) [6]

Following Braun and Clarke’s [56] process for reflexive thematic analysis, our first step was to familiarize ourselves with the data, in which the first two authors (EO and SS) initially read all of the articles. Upon re-reading, both authors took note of passages that stood out and identified patterns shared between the articles (e.g., the aims of the studies and the conclusions that were drawn based on GAMT outcomes). Subsequently, we uploaded the articles into MAXQDA (version 2022) to begin coding. We primarily utilized a deductive coding approach [56, 76]. Informed by trans negativity as a theoretical lens to interpret and extract meaning from the data, we were particularly attentive to cues in the articles indicating a sentiment of “improvement” vis-a-vis GAMT outcomes. However, we did not have an a priori codebook, thus we also used inductive coding by employing open codes that pertained to potentially relevant themes in the text. The first two authors independently coded the designated sections of the articles and labeled relevant content. After the initial coding, they compared their code list and, upon consensus, identified initial themes and subthemes. Multiple rounds of coding were performed to capture all significant themes. We further developed and reviewed these themes with all authors, and once inconsistencies were resolved through discussion, we generated a coding tree along with a final set of themes. Lastly, EO and SS reviewed the relevant quotes for each theme and created a table of exemplar quotes (see Table 2) which was later reviewed and agreed upon by all authors.

**Table 2:** Descriptions of themes and exemplary quotes

Theme	Theme Description	Quotes
*Doing Bad: Experiencing Distress Before GAMT*	This theme captures the emotional, psychological and physical suffering of TGD youth due to gender dysphoria. This suffering is often described as the object of treatment in these articles and manifests in various forms, including dysphoria, anxiety, and depression. The notion of distress (which manifests in different forms) is a key metric for measuring, treating, and evaluating the “effectiveness” of GAMT.	“GD refers to the distress a person may experience when an incongruence exists between one’s sex assigned at birth and one’s experienced gender identity” (69 p302) “Gender-affirming hormones (GAH; estrogen or testosterone) are administered to help alleviate the distress associated with GD” (69 p302) “These individuals have a high prevalence of body image dysphoria, depression and suicidal ideation” (70 p1) “Adolescence is a particularly difficult time for trans-gender persons who experience the development of secondary sexual characteristics that are incongruous with their gender identity, and is associated with a high prevalence of depression and suicidal thoughts and gestures” (70 p1) “As reported in most clinical research among transgender adolescents, the overall psychosocial health of this cohort is impaired at baseline” (21 p1756) “Gender dysphoria (GD) […] is associated with considerable distress or impairment in social, school or other important areas of functioning” (22 p2) “GD refers to this stressful condition resulting in clinically significant distress or impairment in important areas of functioning” (75 p2206) “Some adolescents, who have shown an extreme pattern of cross-gender identification from their earliest years, suffer deeply from the fact that they cannot be open about their gender feelings” (77 pp263-264) “Knowing that they will have to await treatment for many years engenders feelings of hopelessness and slows down their social, psychological, and intellectual development” (77 p264) “They have to cope with adverse consequences of living with a self-concept that is never socially acknowledged or reinforced. In such cases, early treatment would prevent much unnecessary, suffering” (77 p264) “Once these young persons, who are already experiencing con-siderable distress over their gender identity, undergo the pubertal development of their biological sex, their psychological well-being deteriorates significantly” (75 p2207) “Transgender and nonbinary (TNB) youths are disproportionately burdened by poor mental health outcomes, including depression, anxiety, and suicidal ideation and attempts” (74 p2) “Unfortunately, these young people face a range of mental health disparities, including elevated rates of anxiety, depression, and suicide attempts” (72 p2) “Adolescents referred to specialized gender identity clinics have prevalence rates of depression ranging from 12–58% and for anxiety 16–24%” (73 p700) “… found more behavioral and emotional problems in transgender youth compared with the normative samples of these measures” (73 p700) “… they have a gender identity problem” (77 p263) “… the extreme gender identity disorder, called transsexualism” (77 p265) “… with regard to the reduction of GD, the effectiveness of these requirements is not evidence-based” (71 p633)
*Moving Toward a Static Gender Identity and Binary Presentation*	This theme examines the concept of gender transition as a linear, unidirectional process, characterized by a clear beginning and end point, reflecting a binary understanding of gender. In these articles, adolescence is perceived as a critical period for gender identity development. Gender transition is then framed as being completed in (young) adulthood, with an expectation that gender identity stabilizes (suggesting that adulthood brings a sense of permanence). This theme emphasizes gender transition as a linear path within a binary framework (i.e., moving from “man” to “woman” or vice versa).	“… identity development during adolescence is in progress and consolidates only later in adulthood [18], highlighting the need to weigh affirmative treatment practices against a developmental perspective of adolescent identity development during clinical decision making” (21 p1756) “Interventions include psychosocial support, therapy and medical or surgical interventions to align the body with the identified gender” (22 p2) “During the real-life test applicants have to live full-time in the desired gender role. Thus they can discover whether they are able to pass as someone of the opposite sex and experience all advantages and disadvantages of the new situation. Depending on the situation, the role change may occur gradually or at once. (77 p265) “Preventing the development of a body contrary to the experienced gender, puberty suppression allows GD adolescents to experience a smooth transition into their desired gender role” (75 p2212) “…although in most prepubertal children GD will desist, onset of puberty serves as a critical diagnostic stage, because the likelihood that GD will persist into adulthood is much higher in adolescence than in the case of childhood GD” (8 p697) “…participants further along in their transition (higher ITPS) had more subjective positive experiences of receiving TRC… (71 p641) “It is also likely that adolescents with less extreme or more fluctuating cross-gender identities do not pursue SR so early in life. (31 p480) “…clinicians might want to take special notice of MFs who report inconsistencies in past and present gender dysphoria…” (78 p98) “There may also be sex differences related to the social aspect of medical transition. For instance, compared with transgender girls/women, it may be easier for transgender boys/men to integrate socially because of clear vocal changes (i.e., voice deepening) and facial hair growth, which are traditionally seen as indicators of one’s gender” (69 p303) “…adolescents who had undergone a full social transition” (21 p303) “…given that GnRHa does not change the body in the desired direction, but only temporarily prevents further masculinization or feminization” (22 p20). “Strong feelings of belonging to the opposite sex and corresponding behavioural manifestations have been reported as beginning as early as 2 to 3 years of age” (77 p263) “If an adolescent continues to pursue GR, arresting the development of secondary sex characteristics results in a lifelong advantage of a convincing physical appearance congruent with the desired gender role” (80 p2277) “The phenomenon of transsexualism refers to individuals who are born with the normal sexual characteristics of one sex, but have the irrefutable conviction of belonging to the other” (78 p89) “GAH treatment may be a relief to many transgender youths due to both the knowledge that the “correct” hormones are present and the alignment of physical appearance with gender identity” (76 p2) “Early gender dysphoria has been associated with early-onset transsexualism and favourable SR outcome” (78 p98) “Early gender dysphoria has been associated with early-onset transsexualism and favourable SR outcome” (78 p98) “These disparities are generally thought to be due to two processes: gender minority stress and dysphoria related to one’s body developing in ways that are incongruent with one’s gender identity (i.e., a person’s psychological sense of their own gender)” (72 p2) “… we doubt that the reported cases reflect a complete and stable (re)establishment of a gender identity corresponding with genital sex in persons with a lifelong and extreme cross-gender identity” (77 p264) “Despite the fact that developing evidence suggests that cognitve and affective cross-gender identification, social role transition, and age at assessment are related to persistence of childhood GD into adolescence, predicting individual persistence at a young age will always remain difficult” (8 p703) “… according to observers, their appearance better matched the new gender” (78 p96) “The chance of making the wrong diagnosis and the consequent risk of postoperative regret is therefore felt to be higher in adolescents than in adults, as a consequence of the developmental phase itself” (77 p263) “SRS has resolved the patients’ gender identity problem and enabled them to live in the new gender role in quite an inconspicuous way” (77 p269)
*Doing Better: Overall Improvement After GAMT*	This theme highlights the improvements on various registers (global functioning, body (dis)satisfaction, mental health, psychosocial health, psychosocial functioning, social functioning, quality of life, well-being, psychological functioning, decreased gender dysphoria, decreased depression, decreased suicidality) that are measured as outcomes from GAMT, signifying the “effectiveness” of the care.	“Our findings also support the notion that transgender people tend to have more positive life experiences when they receive gender-affirming care” (69 p308) “Further research is needed to statistically assess pre- and post-intervention differences as well as to identify which treatment path fits which adolescent best in order to achieve the best psychosocial health outcomes” (21 p1765) “An alleviation of gender dysphoria can be expected to be closely associated with improvement in other areas of life, such as psychological, social, and sexual functioning” (77 p266) “Even adolescent applicants who are functioning well will need a lot of guidance through the process of sex reassignment. However, provided they manage to pass SRS without problems, they have a lot to gain. They can catch up with their peers and devote their attention to friendships, partnership, and career” (77 p270) “Psychological functioning improved steadily over time, resulting in rates of clinical problems that are indistinguishable from general population samples (eg, percent in the clinical range dropped from 30–7% on the YSR/ASR30) and quality of life, satisfaction with life, and subjective happiness comparable to same-age peers” (8 p702) “Our second aim was to examine how transgender youth body dissatisfaction, depression, and anxiety symptoms change over the first year of receiving gender-affirming hormone therapy. We anticipated improvements in each of these domains but did not have any a priori hypotheses regarding which domains would demonstrate the greatest improvements” (79 p2) “Access to GAH during adolescence appears to be related to more favorable mental health outcomes” (72 p11) “Previous studies have shown that only GR consisting of CSH treatment and surgery may end the actual gender dysphoria” (80 p2281) “This suggests that some internalizing symptoms may be related to improvements in body image dissatisfaction, likely in response to the knowledge that the “correct” hormone is in their body and the physical effects of T” (76 p6) “We hypothesized that (a) suicidality will decrease between pretest and final assessment with the administration of GAH and (b) general well-being will improve between pretest and final assessment with the administration of GAH” (69 p303) “Do gender dysphoric youth improve over time with medical intervention consisting of GnRHa, CSH, and GRS? […] Finally, do young people who report relatively greater gains in psychological functioning also report a higher subjective well-being after gender reassignment?” (8 p697) “We hypothesized that the T treated group would experience fewer anxiety and depression symptoms, and less suicidality; higher satisfaction with body image” (76 p2) “We hypothesized that access to GAH during both early and late adolescence would be associated with more favorable mental health outcomes reported in adulthood, when compared to desiring but never accessing GAH” (72 p3) “We hypothesized a poor general functioning at baseline, an improvement after psychological support, and a further improvement after the beginning of the GnRHa” (75 p2207) “… added as important outcome measures objective and subjective well-being (often referred to as “quality of life”), that is, the individuals’ social life circumstances and their perceptions of satisfaction with life and happiness” (8 p697) “Whereas some studies show that poor surgical results are a determinant of postoperative psychopathology and of dissatisfaction and regret all young adults in this study were generally satisfied with their physical appearance and none regretted treatment” (8 p701) “Overall, the evidence suggests that youth who received GAH and gender confirmation surgery (GCS) for gender dysphoria experience a corresponding alleviation of the dysphoria and overall improved well-being and mental health outcomes” (69 p302) “The findings contribute to a growing literature supporting the hypothesis that transgender adolescents and adults benefit from GAH in terms of quality of life and psychological functioning” (69 p308) “These partially reversible/irreversible treatments aim to align the individuals’ physical appearance with their internal gender identity and have been shown to improve the patients’ psychosocial well-being” (75 p2212) “Results of this study provide first evidence that, after CSH and GRS, a treatment protocol including puberty suppression leads to improved psychological functioning of transgender adolescents.While enabling them to make important age-appropriate developmental transitions, it contributes to a satisfactory objective and subjective well-being in young adulthood” (8 p703) “Recent research points to gender affirmation being the appropriate care for youth GD, when indicated by a thorough assessment process, as trans adolescents are likely to experience improvements to general mental well-being through social and/or medical transition” (71 p633) “Our preliminary results show negative associations between depression scores/suicidal ideation and endocrine intervention, while quality of life scores showed positive associations with intervention, in transgender youths over time in the US” (70 p4) “Symptoms of general anxiety, social anxiety, depression, and suicidality were all lower in the treated than untreated group” (76 p6) “The primary results indicate that transgender adolescent boys undergoing T treatment display lower levels of self-reported anxiety, depression, and suicidality relative to a similar group of adolescents not undergoing GAH therapy. T treatment was also associated with improvements in body image satisfaction” (76 p7) “Participants’ suicidality scores had significantly decreased following administration of GAH” (69 p307) “Ultimately, we may infer from our findings that GAH is associated with less suicidality and greater well-being for all youth” (69 p308) “For each time period of GAH initiation examined (early adolescence, late adolescence, and adulthood), access to GAH was associated with lower odds of past-year suicidal ideation and past-month severe psychological distress” (72 p10) “When it comes to satisfaction with TRMI, the physical effects, particularly as a result of GAH or GAS, seemed to be of paramount importance for adolescents. This is in line with studies showing that medical transition has positive effects on young trans individuals who began transition in adolescence, including decreases in GD and improvements in psychosocial functioning (ie, decrease in depression and anxiety)” (71 p641) “In the group receiving puberty suppression, the externalizing problems (YSR/ASR) score and mental and physical health-related quality of life scores (Kidscreen/SF-8) were within the norm, and clinicians’ ratings of global functioning (CGAS) indicative of good functioning levels at follow-up” (21 p1763) “Moreover, puberty suppression was associated with a further improvement in global functioning. Finally, global functioning improved steadily over time in GD adolescents receiving both psychological support and GnRHa” (75 p2212) “Our results suggest that endocrine intervention is associated with improved mental health among transgender youth” (70 p3) “… participants who accessed GAH earlier had better mental health outcomes, including lower odds of past-year suicidal ideation, past-month severe psychological distress, past-month binge drinking, and lifetime illicit drug use” (72 p11) “Clinicians and advocates working with transgender youth and their families can cite these data as support that GAH is associated with improved psychological outcomes among transgender youth” (69 p308) “The findings contribute to a growing literature showing that transgender adolescents and adults benefit from GAH in terms of improved quality of life and psychological functioning” (69 p309) “This is the first prospective study showing that psychological functioning of adolescents diagnosed with GID had improved in many respects after an average of nearly 2 years of GnRHa use. Adolescents showed fewer behavioral and emotional problems, reported fewer depressive symptoms, feelings of anxiety and anger remained stable, and their general functioning improved” (80 p2281) “This translates into an improvement in many aspects of their psychosocial functioning, such as mood improvement and school integration [2, 9]. Consistently, these results underline the importance of puberty suppression for GD adolescents’ well-being” (75 pp2212) “The present study, together with this previous research [2], indicate that both psychological support and puberty suppression enable young GD individuals to reach a psychosocial functioning comparable with peers” (75 pp2213)
*Absence of Regret*	This theme highlights the importance placed on minimizing the chances of regret in GAMT for adolescents, aligning with the idea that gender identity can be fluid during adolescence and may solidify in young adulthood. Avoiding regret is commonly seen as essential for achieving “effective” GAMT outcomes for adolescents.	“The chance of making the wrong diagnosis and the consequent risk of postoperative regret is therefore felt to be higher in adolescents than in adults, as a consequence of the developmental phase itself.” (77 p263) “Whereas some studies show that poor surgical results are a determinant of postoperative psychopathology and of dissatisfaction and regret, [37, 38] all young adults in this study were generally satisfied with their physical appearance and none regretted treatment.” (8 p701) “Adolescents and young adults rarely regret or stop TRMIs, provided they fulfill the criteria for a GD diagnosis and their readiness for treatment is sufficiently assessed” (71 p633) “With respect to prevailing uncertainties when it comes to treatment of trans youth and desires of HCPs to avoid mis-diagnoses, […] an important finding is that no adolescents and young adults in the present study regretted TRC at the time of follow-up, mirroring other studies that determined no regret of GnRHa administration or GAH and GAS” (71 p641) “One of the main objections of profes-sionals against a start of the SR procedure before 18 years is the risk of postoperative regrets” (31 p472) “…postoperative regret or any other unfavorable result is a matter of serious concern” (31 p472) “Above all, no one expressed feelings of regret concerning the decision to undergo SRS” (31 p472) “Considering the invasive and irreversible treat-ment of SR, it is imperative to try and prevent post-operative regret. This requires the identi-fication of predictors of regret or poor post-operative functioning” (78 p90)

### Positionality statement

A central aspect of reflexive thematic analysis is acknowledging that researchers’ positions influence the research process, inviting researchers to critically reflect on their subjectivity and positionality [55]. We, the authors, comprise a mix of junior (EO and SS) and senior researchers (KG, DD, and AV). The first two authors (EO and SS) are PhD candidates with a background in Gender Studies and Sociology. KG is a trained ethicist and psychiatry resident with clinical and research experience in GAMT. DD is an academic researcher trained in social and medical psychology whose work primarily focuses on members of marginalized groups. AV is a child psychiatrist and senior researcher with extensive clinical and research experience on adolescent transgender care and served as a co-author on the SOC8 Adolescents chapter. All authors are currently affiliated with the Center of Expertise on Gender Dysphoria (CEGD) in Amsterdam, the Netherlands. The authors represent varying gender identities and sexual orientations, including trans, queer, and cis.^6^ One of the authors has experience with accessing GAMT in the Netherlands as a young adult. All of the authors are White, highly educated, live in a high-income country, have academic affiliations, and hold Western citizenship. While our professional and personal backgrounds offer different perspectives on GAMT for adolescents, we acknowledge the limitations in our insight into specific challenges at various intersections of TGD identities in terms of race, class, and (dis)ability.

As authors, we understand the precariousness of this care practice, experiencing it as both recipients and providers of GAMT. Like the authors of the SOC8, we acknowledge the progress made in the provision of GAMT and its significant potential in enhancing the overall physical and psychological health of TGD individuals. We recognize the risks associated with subjecting GAMT outcomes to critical scrutiny, especially given the ongoing limitations on its provision in several countries. Many proponents of GAMT for adolescents have responded to these care restrictions by emphasizing “positive” research outcomes (i.e., improved well-being, low regret rates etc.) which is both valuable and necessary. While this article takes a different approach, aiming to build upon existing work, our intention is not to question the value of GAMT for TGD youth. Instead, we aim to question claims that GAMT must necessarily result in “effective” outcomes in order to be considered legitimate and essential care. Our intention, then, is not to undermine the legitimacy of GAMT but rather, echoing Malatino [2] and Saketopoulou and Pellegrini [77], to articulate the complex and ambivalent experiences of gender transition. Our interest, as such, lies in making space for diverse perspectives on what GAMT *ought* to do, moving beyond normative notions of improvement that might limit the diverse experiences and needs of TGD individuals.

## Results

### Overview of measures

Throughout these 16 cited sources, we identified 44 different measures in total that were used to quantitatively assess and evaluate the “effects” of GAMT for adolescents (see Table 1). These measures assessed constructs which we have classified under the following categories: gender dysphoria and body dissatisfaction, psychological functioning, global functioning, social functioning, quality of life, and satisfaction with care. Many of these measures pertained to psychological functioning (20 of the 44 measures), and only four of these measures assessed gender dysphoria and body dissatisfaction. In terms of how frequently authors measured each of these constructs, psychological functioning was most often measured, assessed in 14 of the 16 studies. Gender dysphoria and body dissatisfaction was the second most frequently assessed construct (*N* = 9), followed by social functioning (*N* = 6), quality of life (*N* = 6), global functioning (*N* = 4), and satisfaction with care (*N* = 3).

### Reflexive thematic analysis

Using trans negativity as a theoretical lens [1–3], we analyzed the cited literature in the “Research Evidence” section of the SOC8 Adolescents chapter and identified four interrelated themes that are used to substantiate “effective” treatment outcomes for GAMT for adolescents. These themes pertain to (1) *doing bad: experiencing distress before GAMT*, (2) *moving toward a static gender identity and binary presentation*, (3) *doing better: overall improvement after GAMT*, and (4) the *absence of regret*. Although these four themes do not all directly describe treatment outcomes of GAMT, together they represent a prevalent narrative throughout the literature that exemplifies what GAMT is expected to treat, as well as how an “effective” GAMT trajectory is commonly described.

We present the themes in this particular order to emphasize the teleological narrative commonly portrayed in medical literature, which we aim to challenge: a progression from “doing bad” to “doing better,” ultimately leading to an overall improvement in the individual’s functioning and well-being. This narrative also suggests that gender identity is potentially malleable during adolescence but tends toward a stable endpoint, solidifying into a static identity in young adulthood. Furthermore, it suggests movement within a binary understanding of gender, portraying gender transition as a chronological process with a clear beginning and endpoint. Such a framing of gender transition upholds the dominant understanding of GAMT as a linear process with a stable, teleological outcome. Central to these themes is a pervasive “logic of improvement,” implying that GAMT is “curative,” supposedly guiding TGD individuals from “doing bad” to “doing better” in a linear, teleological manner. In the subsequent sections, we will discuss how each theme reveals the underlying expectations that define the perception of GAMT as “effective,” reflecting broader discourses on the objectives and outcomes of GAMT for adolescents.

### Theme 1. Doing bad: experiencing distress before GAMT

The first theme encapsulates the profound physical, psychological, and psychosocial distress experienced by TGD adolescents. Throughout the cited literature in the SOC8 “Research Evidence” section, distress is frequently identified as the primary “target” of GAMT, suggesting that one of its key objectives is to alleviate or even resolve this distress. Furthermore, this distress is often attributed to gender dysphoria experienced by TGD youth. Although the Body Image Scale (BIS) and the Utrecht Gender Dysphoria Scale (UGDS) were commonly used to measure and evaluate body (dis)satisfaction and (the intensity of) gender dysphoria respectively, a substantial proportion of the cited articles (7 out of 16) did not use either of these measures [19, 70–75]. Instead, they focus on assessing constructs such as global psychosocial functioning and psychological well-being (see Table 1).

In this cited literature, gender dysphoria is commonly characterized as an incongruence between the individuals’ body and their identity: “a conflict between a person’s physical or birth-assigned sex and the gender with which that person identifies and the wish to receive medical interventions that modify the body” (21 p1). Authors describe that gender dysphoria is “often accompanied by psychological distress and a persistent strong desire for social and physical gender changes” (71 p633). Notably, in many countries, a diagnosis of Gender Dysphoria *accompanied by distress* is a requirement for accessing GAMT [35]. If gender dysphoria refers to a persons’ distress, then a certain level of distress (i.e., “doing bad”) must be experienced to receive GAMT.^7^ This distress, however, remains ambiguous in how it is defined and identified.

Although few authors acknowledge the diversity in TGD peoples’ experiences, Allen et al. (69 p302) note that “transgender people have varying degrees of GD; some have none at all,” acknowledging that “distress” can manifest differently for different people. The distress experienced by TGD adolescents often extends beyond the physical discomfort associated with gender dysphoria to include psychological and psychosocial challenges that affect social interactions, school, and other critical aspects of life [20]. All 16 studies highlight the psychological effects of this distress, and the risk of this distress worsening if GAMT is withheld. As Costa et al. (75 p2207) state:

Despite many years of psychotherapy the [gender dysphoria] of most adolescents does not often abate. Rather, once these young persons, who are already experiencing considerable distress over their gender identity, undergo the pubertal development of their biological sex, their psychological well-being deteriorates significantly.

As the literature describes, TGD youth are disproportionality burdened by poor mental health outcomes before receiving GAMT, including depression, internalizing disorders, behavioral problems, anxiety, and suicidal ideation and attempts [66, 70, 72]. Achille et al. (70 p3) report that this distress becomes pronounced during adolescence, a period characterized as “a particularly difficult time for transgender persons who experience the development of secondary sexual characteristics that are incongruous with their gender identity, and is associated with a high prevalence of depression and suicidal thoughts and gestures.”

The intense distress experienced by TGD adolescents is highlighted by Cohen-Kettenis and Van Goozen (77 p264), who note that TGD individuals showing “an extreme pattern of cross-gender identification from their earliest years, suffer deeply.” Consequently, delaying initiation of GAMT “engenders feelings of hopelessness and slows down their social, psychological, and intellectual development,” suggesting that “early treatment would prevent much unnecessary suffering” throughout (young) adulthood (77 p264). By emphasizing the negative experiences of TGD youth including physical, psychological, and psychosocial distress, specifically *before* receiving GAMT, these authors justify the benefits of and necessity for gender-affirming medical interventions, emphasizing the urgency for appropriate and effective support for TGD adolescents.

However, an underlying uncertainty around the “effectiveness” of early intervention remains present throughout the cited literature. For instance, De Vries et al. (8 p697) acknowledge that “despite the apparent usefulness of puberty suppression, there is only limited evidence available about the effectiveness of this approach.” Almost all articles (15 out of 16) highlight the limited evidence-base supporting the provision of GAMT for TGD adolescents [6, 19, 20, 29, 62, 64–74]. Becker-Hebly et al. [19] note that the numbers of studies providing evidence that both puberty suppression and GAH therapy can improve multiple, but not always all, aspects of psychosocial health remains small. Furthermore, critics of GAMT for adolescents express concerns that suppressing puberty may even negatively affect psychological functioning [65]. As such, the ambiguity surrounding the “effectiveness” of (early) GAMT for TGD adolescents stresses the complexity of this care practice. This uncertainty appears to arise from both the limited evidence base and the ambiguous ‘nature’ of the distress required to receive GAMT, reflecting broader socio-political debates about the object and objectives of GAMT for youth – that is, what GAMT aims to “treat” and “achieve” in the first place [48, 78, 79].

### Theme 2. Moving toward a static gender identity and binary presentation

The second theme we identified concerns the linear and predetermined nature of gender identity development and gender transition. Throughout the cited literature in the SOC8 section, both gender identity development and gender transition tend to be depicted as teleological, unidirectional processes.

Drawing from the cited literature, it appears that the concept of a linear gender transition toward a fixed endpoint is rooted in the idea that adolescence is a crucial period for the development of gender identity: “identity development during adolescence is in progress and consolidates only later in early adulthood” (21 p1756). Through this framing, gender identity development is perceived as being potentially fluid early in life but is eventually expected to stabilize in a binary way in (young) adulthood. As the authors highlight, a stable, persisting and (often) binary gender identity^8^ thus becomes a prerequisite for receiving GAMT: “clinicians might want to take special notice of MFs [male to females] who report inconsistencies in past and present gender dysphoria” (78 p98). In this context, an unstable gender identity is framed as problematic by Smith et al. (78 p98):

The contradicting presence of more gender dysphoria in childhood but less at application [of GAMT] should alert the clinician when assessing eligibility. This inconsistency may reflect confusion about development, an (unconscious) exaggeration of history if current feelings are not clear-cut, or a conscious effort to mislead the clinician.

However, De Vries et al. (8 p703) note that the persistence of a certain gender identity is not necessarily guaranteed:

Despite the fact that developing evidence suggests that cognitive and affective cross-gender identification, social role transition, and age at assessment are related to persistence of childhood [gender dysphoria] into adolescence, predicting individual persistence at a young age will always remain difficult.

In this context, De Vries et al. [6] suggest that gender identity might be malleable in childhood, meaning that a more fluid gender identity in childhood might desist and become more fixed into adolescence. However, De Vries et al. (80 p2281) maintain that TGD youth should exhibit a consistent and persistent experience of gender dysphoria – manifested as distress – in order to receive GAMT; “young adolescents who [have] been carefully diagnosed show persisting gender dysphoria into late adolescence or young adulthood” and can benefit from GAMT. The cited literature, which adheres to the SOC8 eligibility criteria for the provision of GAMT for adolescents, imply an underlying assumption that TGD adolescents who would benefit from GAMT can be identified through careful clinical evaluation. Those who are determined to be experiencing persistent and identifiable dysphoria are more likely to access GAMT to “effectively” align the body with the identified gender and to relieve the young individual from suffering.

Suppressing puberty through gonadotropin releasing-hormone analogues (GnRHa) is an important aspect of medical intervention for TGD youth, but its implications are framed differently by various authors. This highlights an important question – and a point of tension – surrounding GAMT for youth: whether puberty suppression allows time for TGD youth to pause and make decisions, or whether it is a first step that will inevitably lead to further treatment. For instance, Carmichael et al. (22 p20) describe the provision of GnRHa as a “pause,” pointing out that “GnRHa does not change the body in the desired direction, but only temporarily prevents further masculinization or feminization.” In this view, suppressing puberty through GnRHa is seen as a temporary measure providing TGD adolescents with additional time to explore their gender identity without the distress caused by the development of secondary sex characteristics, thereby providing time to assess the stability of their gender identity *before* proceeding with more irreversible medical interventions. Conversely, Costa et al. (75 p2212) argue that puberty suppression allows TGD adolescents “to experience a smooth transition into their desired gender role.” In this context, the authors frame puberty suppression as a first medical step toward achieving a binary gender expression rather than merely a pause for contemplation before continuing with GAMT interventions.

Furthermore, this “smoothness” underlies a key normative assumption in the provision of GAMT for youth; as De Vries et al. (80 p2277) articulate, “if an adolescent continues to pursue GR [gender reassignment], arresting the development of secondary sex characteristics results in a lifelong advantage of a convincing physical appearance.” Cohen-Kettenis and Van Goozen (77 p269) also note that gender-affirming surgeries enabled TGD individuals “to live in the new gender role in quite an inconspicuous way.” These examples highlight the concept of passing – “successfully” being perceived as the desired (binary) gender – and further construct gender transition in a binary manner. As such, these authors frame “effective” GAMT as not only alleviating the aforementioned “distress,” but also as resulting in a congruent, “inconspicuous” binary gender presentation. Allen et al. (69 p303) note that the ability to “pass” might depend on sex differences:^9^ “compared with transgender girls/women, it may be easier for transgender boys/men to integrate socially because of clear vocal changes (i.e., voice deepening) and facial hair growth, which are traditionally seen as indicators of one’s gender.”

These examples illustrate the discourse surrounding GAMT as a teleological process, progressing through distinct stages toward a stable, congruent, and binary gender identity and gender presentation. This portrayal emphasizes a normative understanding of gender development and transition, with a clear beginning of gender dysphoria early in life, persisting throughout adolescence and stabilizing in young adulthood. Such as framing restricts the recognition that fluidity in gender identity is neither abnormal nor pathological [30, 80, 81]. Throughout the literature, the concept of “smoothness” underscores the narrative of a “successful” transition, where individuals seamlessly align with the identified and presented (binary) gender. This notion of a linear and binary transition pathway is closely tied to the expectation of achieving to “pass,” indicating a “successful” transition [82].

### Theme 3. Doing better: overall improvement after GAMT

The third theme describes the multi-faceted improvements observed across various measures of TGD youths’ functioning and well-being following GAMT. These improvements include treatment outcomes spanning a wide range of constructs, from *decreased* gender dysphoria and body dissatisfaction, to *improved* global functioning, psychosocial health, mental health, and overall well-being and quality of life.

In nine studies, the alleviation and/or resolution of gender dysphoria, measured through either the BIS, the UGDS, or both, was characterized as a critical result of GAMT [6, 20, 29, 64–69]. In some studies, gender dysphoria was described as being “cured” after GAMT; Smith et al. (31 p479) report that “gender dysphoria had disappeared after treatment” and, referring to previous studies, De Vries et al. (80 p2281) note that GAMT indeed may “end the actual gender dysphoria.” Although gender dysphoria was considered a primary treatment outcome in these texts, questions remain as to whether it *should* be the main – or only – treatment target. De Vries et al. (8 p297) reason that, “after all, treatment cannot be considered a success if [gender dysphoria] resolves without young adults reporting they are healthy, content with their lives, and in a position to make a good start with their adult professional and personal lives.” Indeed, in each of the studies, gender dysphoria was not the only reported treatment outcome to evaluate GAMT.

Treatment outcomes of GAMT related to psychosocial functioning reportedly led to enhancements in many aspects of TGD youths’ functioning, “such as mood improvement and school integration” (75 p2212). De Vries et al. (8 p702) report that TGD individuals’ psychological functioning improved, and quality of life, satisfaction with life, and subjective happiness became comparable to cisgender peers of the same age after GAMT. As such, De Vries et al. (8 p702) state that GAMT provides “formerly gender dysphoric youth the opportunity to develop into well-functioning young adults.”

The sentiment that GAMT can lead to a wide variety of improvements, i.e. “doing better” in various domains is a key argument in this literature as to how GAMT can be “effective” or beneficial for TGD youth: “a treatment protocol including puberty suppression leads to improved psychological functioning of transgender adolescents. While enabling them to make important age-appropriate developmental transitions, it contributes to a satisfactory objective and subjective well-being in young adulthood” (8 p703). Furthermore, Cohen-Kettenis and Van Goozen (77 p270) note that, provided they manage GAMT without problems, TGD adolescents have a lot to gain throughout their life course after treatment: “they can catch up with their peers and devote their attention to friendships, partnerships, and career.” Allen et al. (69 p308) echo these broad improvements in well-being: “transgender people tend to have more positive life experiences when they receive gender-affirming care.”

Although many authors highlight the various improvements in TGD adolescents’ lives following GAMT, providing evidence that they are “doing better,” various studies also report minimal or no improvements after GAMT. This highlights a tension in the narrative that GAMT inevitably leads to more positive life experiences and general improvements. For instance, while psychosocial health outcomes of TGD individuals were generally closer to the population norm following GAMT, Becker-Hebly et al. (21 p1763) note that “not all psychosocial health problems seemed to be resolved;” baseline difficulties persisted throughout the follow-up period for TGD adolescents receiving puberty suppression.

Similarly, Carmichael et al. [20] observe that GnRHa treatment did not bring either measurable benefits or harm to psychological function in TGD adolescents, concluding a lack of significant changes in psychological function, quality of life, or the degree of gender dysphoria. Additionally, Kuper et al. [67] acknowledge that environmental stresses may not improve after GAMT, and could potentially worsen, especially if they increase the youth’s visibility as a TGD individual. As Smith et al. (78 p97) assert, “alleviation of the gender problem is not equivalent with an easy life.” Indeed, as Turban et al. (72 p11) emphasize, TGD individuals continue to “face a range of other psychosocial stressors that contribute to chronic minority stress, including but not limited to employment discrimination, lack of safe access to public facilities, and physical violence.” These systemic, socio-political factors can greatly impact TGD individuals’ quality of life, well-being, and in turn, the persistence of *negative affect* [75]. The idea that GAMT cannot remedy all psychosocial stressors is echoed by Tordoff et al. (74 p2): “initiation of GAHs may present new stressors that may be associated with exacerbation of mental health symptoms early in care, such as experiences of discrimination associated with more frequent points of engagement in a largely cisnormative healthcare system.”

These examples point to the idea that GAMT might not always result in “doing better.” To us, this raises the question of whether GAMT can be considered an “effective” treatment even if it does not consistently lead to improvement. Authors of the reviewed papers note that these outcomes alone might not be representative of the broader context contributing to TGD individuals’ state of well-being. De Vries et al. (8 p702) report “positive” treatment outcomes but acknowledge that this was likely not due to GAMT alone: “the positive results may also be attributable to supportive parents, open-minded peers, and the social and financial support (treatment is covered by health insurance) that gender dysphoric individuals can receive.” They emphasize that healthcare providers in the field “should realize that it is not only early medical intervention that determines this success, but also a comprehensive multidisciplinary approach that attends to the adolescents’ [gender dysphoria] as well as their further well-being and a supportive environment” (8 p703).

### Theme 4. Absence of regret

The fourth theme – related to the topic of regret – permeates much of the discussion on treatment outcomes in the cited literature, influencing considerations of eligibility for GAMT, decision-making processes during GAMT, and the criteria for what is considered an “effective” outcome of GAMT.

Several studies discuss regret and the importance of considering and preventing the risk of regret [6, 19, 29, 64, 68, 74]. As Becker-Hebly et al. (21 p1765) assert, this concern is especially pertinent regarding “irreversible” interventions that could result in “possible regret over the body or surgical results.” According to Cohen-Kettenis and Van Goozen (77 p263), regret is particularly important to consider in the context of GAMT for youth: “the chance of making the wrong diagnosis and the consequent risk of postoperative regret is [...] felt to be higher in adolescents than in adults, as a consequence of the developmental phase itself.” This underscores the idea that gender identity in childhood and adolescence is more malleable than in adulthood, potentially increasing the risk of making “wrong” treatment decisions during this development period.

When evaluating regret as an outcome of GAMT, five studies highlight that participants reported minimal or no feelings of regret regarding GAMT. This lack of regret is generally portrayed as a positive and important result, reinforcing the idea that feelings of regret are an “unfavorable result” and “a matter of serious concern” (31 p472). Similar to Cohen-Kettenis and Van Goozen [64], Smith et al. (78 p90) emphasize the importance of avoiding postoperative regret: “considering the invasive and irreversible treatment of SR [sex reassignment], it is imperative to try and prevent postoperative regret. This requires the identification of predictors of regret.” Nevertheless, they report that “the vast majority expressed no regrets about their SR [sex reassignment]” (78 p96). Mentioning that poor surgical outcomes can lead to psychopathology and dissatisfaction, De Vries et al. (8 pp700-701) highlight that all young adults in their study were generally satisfied and that “none of the participants reported regret during puberty suppression, CSH [cross-sex hormone] treatment, or after GRS [gender reassignment surgery].” Nieder et al. (71 p633) seem to interpret the process of assessing TGD youths’ eligibility for GAMT as being intended as a means to prevent the potential of regret in the future: “adolescents and young adults rarely regret or stop TRMIs [transition-related medical interventions], provided they fulfill the criteria for a [gender dysphoria] diagnosis and their readiness for treatment is sufficiently assessed.” An important finding then, Nieder et al. (71 p641) state, “is that no adolescents and young adults in the present study regretted TRC [transition-related care] at the time of follow-up, mirroring other studies that determined no regret of GnRHa administration or GAH [gender-affirming hormones] and GAS [gender assignment surgery].”

The topic of regret serves as a common thread within discussions of treatment outcomes in the cited literature and seems to profoundly influence considerations of eligibility for GAMT and decision-making processes. The possibility of regret is notably highlighted as a critical factor to be addressed to ensure the “effectiveness” of GAMT and feelings of regret are portrayed as “unfavorable,” indicating that the absence of regret is a “positive” result. Authors often seem to equate regret with detransition, portraying both as unambiguously “negative” outcomes, leaving little room for a more nuanced understanding of the diverse ways in which TGD individuals might experience (de)transition [30, 80, 83]. As such, the cited literature stresses minimizing regret as an essential aspect of ensuring the “effectiveness” of GAMT for adolescents.

## Discussion

In this article, we examined what constitutes “effective” GAMT treatment outcomes and how normative assumptions regarding GAMT for adolescents are reflected through these outcomes. Utilizing trans negativity as a theoretical lens, we conducted a theory-informed reflexive thematic analysis of the cited literature in the “Research Evidence” section of the SOC8 Adolescents chapter. Our primary goals were to (1) provide an overview of the treatment outcomes cited in the literature and (2) to elucidate the normative assumptions underlying these outcomes.

In total, we identified 44 different measures which were used to assess GAMT outcomes for adolescents (see Table 1). These measures cover a broad range of constructs, including gender dysphoria and body dissatisfaction, psychological functioning, global functioning, social functioning, quality of life, and satisfaction with care. Notably, many of these measures are used in only a single study, and authors themselves explained and interpreted the use of these measures in varying ways. Despite this overall lack of uniformity in outcome measures, psychological functioning was the most frequently assessed construct across these sources. This highlights the authors’ emphasis on “effective” treatment in terms of improved psychological functioning.

Our theory-informed reflexive thematic analysis revealed four main themes that underpin an “effective” GAMT trajectory and treatment outcomes: (1) *doing bad: experiencing distress before GAMT*, (2) *moving toward a static gender identity and binary presentation*, (3) *doing better: overall improvement after GAMT*, and (4) the *absence of regret*. Ultimately, we found that the cited literature in the “Research Evidence” section of the SOC8 Adolescents chapter tends to portray GAMT as a process that generally follows a movement from “doing bad” to “doing better,” achieving a stable gender identity and avoiding post-treatment regret. Empirical evidence showing pre-transition distress along with physical, psychological, and/or psychosocial improvements post-GAMT, demonstrated through quantitative data, is used to support claims about the “effectiveness” of GAMT for adolescents.

### Justifying the “effectiveness” of GAMT for adolescents: a logic of improvement

Taken together, the SOC8 positions these 16 sources as research evidence to justify the provision of GAMT for adolescents. Citing multiple studies [19, 20, 67, 69, 71–73], the authors of the SOC8 Adolescents chapter assert that “the data consistently demonstrate improved or stable psychological functioning, body image, and treatment satisfaction,” classifying these improvements as “*positive* results of early medical treatment” (1 p46, emphasis added). Although they acknowledge the limitations of the existing studies, such as relatively small sample sizes and varying follow-up periods, they argue that the “emerging evidence base indicates a general improvement in the lives of transgender adolescents” (1 p47). Furthermore, they state that “the data show early medical intervention – as part of broader combined assessment and treatment approaches focused on gender dysphoria and general well-being – can be *effective* and helpful for many transgender adolescents seeking these treatments” (1 p47, emphasis added). Hence, the SOC8 authors use these “positive” empirical results to justify the provision of GAMT for adolescents.

The flip side of the above is that improvement has become a norm that GAMT is required to meet in order to be justified, often operationalized by measurable, beneficial effects on the overall well-being of TGD adolescents. However, our findings indicate ambiguity regarding the objectives of GAMT for adolescents. Should its primary aim be to alleviate gender-related distress, or the improvement of general well-being and functioning in order for it to be justified? While most of the sources cited in the SOC8 highlight the need for more research on GAMT for adolescents, it seems there is not yet consensus on how to evaluate its efficacy, as evidenced by the broad diversity of measures detailed in Table 1.

Furthermore, the (implicit) normative expectation that GAMT should result in improvements across multiple physical, psychological, and psychosocial outcomes risks undermining the provision of this care practice. Indeed, critics often refer to the supposed failure of GAMC to result in improved psychological well-being and psychosocial functioning to question the validity of GAMT:

The significant rate of problematic adaptations, psychiatric symptoms, and self-harm in this youth cohort […] is explained away as merely manifestations of minority stress, with unsubstantiated claims that these mental health problems will resolve with gender transition—and only with gender transition ([84] p115).

Not all psychiatric and psychosocial problems in adolescents displaying gender dysphoria are secondary to gender identity issues and will not be relieved by medical gender reassignment. An adolescent’s gender identity concerns must not become a reason for failure to address all her/his other relevant problems in the usual way (60 p218).

These critiques have far-reaching policy consequences. As Amin [3] notes, legislators have used studies suggesting that GAMT shows insufficient psychological and psychosocial improvement as a basis to outlaw this care for minors. Consequently, research that concludes anything less than unequivocal “effectiveness” of GAMT risks providing critics of this care practice with “ammunition to attack trans medical care” ([85] p345). It is in this context that the force and constraint of the logic of improvement become apparent: “improvement” seems one of the only ways to justify this care practice for TGD adolescents, but comes at the cost of obscuring and rendering invisible more diverse and nuanced experiences of GAMT and risks discrediting this care practice. The latter has serious ethical implications for clinical practice and (shared) decision-making: the logic of improvement risks reproducing (largely implicit) normative images of “straightforward” presentations of gender dysphoria and “good functioning” clients as opposed to “complex” clients with co-occurring mental health problems whose experiences of gender dysphoria are perceived by care healthcare providers as unstable or less credible.

Not only does this logic limit space for more diverse and nuanced experiences, it can also put a strain on the therapeutic relationship between healthcare providers and TGD youth themselves [26, 86–88]. For example, there is a prevalent fear among TGD individuals who want to access GAMT that not showing *enough distress* will impact their eligibility for care [26, 87, 88]. This places further tension on the provider-client relationship; TGD individuals may see their healthcare providers as gatekeepers, hindering honest communication due to a fear that it may jeopardize their care [25, 86]. This medical model can function to push healthcare providers into the role of gatekeepers, who are then expected to navigate the inherent uncertainty involved in this care and prevent any risk of regret [25].

Furthermore, challenging the logic of improvement has significant clinical implications. For example, it becomes imperative for healthcare providers to engage in open conversations with TGD individuals and their families or caregivers about the possibility that GAMT may not lead to the expected or desired outcomes. As discussed earlier, this narrative of transition as “curative” is not limited to medical settings; it is also prevalent within TGD communities. However, framing GAMT as entirely curative may impose unrealistic expectations on both the treatment itself as well as healthcare providers to deliver exclusively “positive” outcomes [30, 80]. Openly addressing and accepting the wide range of potential developments and treatment outcomes – including changes in the individual’s gender identity, treatment preferences, regret, and the possibility of retransition or detransition – will foster a more nuanced and diverse understanding of GAMT, helping TGD youth, their parents or caregivers, and healthcare providers to make well-informed decisions. Taking this approach to GAMT not only relieves the pressure on this form of care to “fix” several aspects of a person’s life but also allows for a more nuanced and realistic understanding of the “effectiveness” of GAMT.

### Moving beyond the logic of improvement

While some cited articles in the SOC8 Adolescents chapter acknowledge that GAMT alone may not lead to improvement in overall well-being and functioning, the prevailing literature implicitly assumes that GAMT is “effective” and justified when a stable gender identity is attained and psychological well-being and psychosocial functioning improve. The teleological nature of this narrative is inherent in the logic of improvement and suggests that there is a measurable endpoint in which GAMT has been “effective” for the individual. The literature cited in the SOC8 Adolescents chapter generally frames TGD adolescents’ lives before GAMT as marked by distress and intense psychological suffering and life after GAMT as characterized by improvement across physical, psychological, and psychosocial registers.

However, this teleological account of transition – resulting in alignment between one’s gender identity and body, alongside improved well-being – risks oversimplifying the often complex and ambivalent experiences of gender transition into a linear narrative of improvement; the expectation that this care could address all aspects of general functioning and well-being is unrealistic. Further, this expectation of gender transition as a step-by-step linear process can harm those undergoing treatment, creating external pressure to follow a specified trajectory [30, 80, 83]. While GAMT often aids in achieving gender congruence and overall improvement, benefiting the lives of young TGD individuals, the justification of this care practice should not be conditional on this logic of improvement. Trans negativity [1–3, 89] challenges the dominant discourse that GAMT must necessarily alleviate distress and lead to improvement in overall well-being and functioning in order to be justified, instead acknowledging that negative feelings often persist after, or even because of, GAMT. As Malatino (4 p26) states, trans negativity challenges the dominant framing of GAMT characterized by a period of distress, followed by an “experience of harmony, good feeling, corporeal comfort, and ease when navigating everyday social interactions.”

In other words, while narratives of improvement can function to justify GAMT, they risk excluding more nuanced and complex experiences. Trans scholars argue that experiences of GAMT are often messier, more ambivalent, and temporally more complex than the binary of “doing bad before GAMT” and “doing better after GAMT” [90–92]. For example, Chu (2 np) notes, “I feel demonstrably worse since I started on hormones,” and mentions increased suicidal ideation after GAMT. Despite feeling worse during her transition, Chu (2 np) states, “transition doesn’t have to make me happy for me to want it [...] Desire and happiness are independent agents.” Consequently, Chu (2 np) argues that the only prerequisite for GAMT should be a demonstration of desire, asserting that “no amount of pain, anticipated or continuing, justifies its withholding” and that GAMT cannot be expected to “maximize good outcomes.” Chu’s perspective contributes an alternative for moving beyond the logic of improvement narrative, and therefore beyond diagnostic prerequisites and “effective” treatment outcomes.^10^ In a similar vein, Amin [3] challenges the normative notion that individuals undergoing GAMT should aspire to happiness and that GAMT inherently leads to this outcome, instead asserting that gender transition should not be expected to eliminate all negative feelings. Rather, negative affective states, such as the experience of regret, are inherent to all lives, including those of TGD individuals [1–3]. As Malatino states, “transitioning doesn’t have to be wholly curative, or even minimally happy-making, in order for it to be imperative” (4 p3).

## Strengths and limitations

Regarding our methodology, it is important to note the strengths and limitations of our work. A strength of our approach is the use of trans negativity as a theoretical lens. Trans studies and biomedical sciences have traditionally existed in separate spheres, limiting the integration of nuanced understandings of GAMT into transgender healthcare. Trans studies broadly, and theorizations of trans negativity specifically, offer a valuable perspective for reimagining transgender healthcare and fostering more nuanced discussions. Our approach of reflexive thematic analysis is inherently subjective; we value moving away from claiming a “neutral” position in this field, making explicit our subjectivities and the positionalities that inform this work. While we believe our positionalities enhanced our analysis, we acknowledge that we potentially missed nuances in the data due to our deductive thematic approach.

## Conclusion

While GAMT *does* often aid in achieving gender congruence and overall improvement, benefiting the lives of young TGD individuals, engaging with trans negativity as a theoretical lens emphasizes that negative feelings can persist post-GAMT. This perspective encourages critical reflection on the normative assumption that GAMT must inevitably lead to “positive” outcomes to justify its provision. Instead of solely focusing on substantiating the “effectiveness” of GAMT with empirical evidence and justifying its provision by showing overall improvement, we should explore how to better support healthcare providers and TGD individuals in navigating negative feelings throughout and post-GAMT. Allowing space for these complex experiences, rather than trying to avoid or mask them, could offer relief for both healthcare providers and TGD adolescents and foster a more honest care environment.

However, questioning the current operationalization of “effectiveness” in GAMT for adolescents raises a critical question: if GAMT does not necessarily require demonstrating improvement to justify its provision, what should its objectives be? In other words, what ethical and philosophical justifications should underpin GAMT for adolescents, and what does *good* GAMT for adolescents entail? As we have seen, the treatment outcomes presented in the SOC8 “Research Evidence” section of the Adolescents chapter primarily rely on the ethical principles of beneficence and non-maleficence, with the provision of GAMT largely justified by empirical outcomes demonstrating its “effectiveness.” Others have proposed alternative ethical frameworks for justifying the provision of GAMT for adolescents; for example, by drawing an analogy to interventions like abortion and birth control [9]. Similar to GAMT, these interventions alter healthy physiological states based on an individual’s fundamental self-conception and desired life path, with their effectiveness measured by how well they help individuals achieve their embodiment goals [9]. In this view, healthcare is provided and justified on the basis of personal desire and autonomy.

While we do not propose a definitive answer to this complex question, we aim to initiate a normative discussion on how the “effectiveness” of GAMT for adolescents should be assessed. One promising approach to achieve this is through participatory action research, which involves TGD youth in the research process to better understand what they find important regarding GAMT and its outcomes [93, 94]. Participatory action research, which has been employed in other areas of medical research, is considered particularly valuable for building community ties and addressing power imbalances within research [95]. While it is important to acknowledge that TGD youths’ preferred outcomes are not monolithic – participatory action research will not yield a single specific outcome to assess the “effectiveness” GAMT – it will provide a more truthful understanding of what matters to TGD adolescents, facilitating conversations about GAMT with youth and supporting healthcare providers and clients in (shared) decision-making.

## Data Availability

No datasets were generated or analysed during the current study.

## Abbreviations

GAMT: Gender-affirming medical treatment
TGD: Trans and gender diverse
SOC: Standards of Care
WPATH: World Professional Association for Transgender Health
GD: Gender dysphoria
BIS: Body Image Scale
UGDS: Utrecht Gender Dysphoria Scale
GAH: Gender-affirming hormones
GnRHa: Gonadotropin releasing-hormone analogues
T: Testosterone
CSH: Cross-sex hormones
TRMI: Transition-related medical interventions
TRC: Transition-related care

## This terminology is outdated and currently considered inappropriate

MF: Male-to-female
SR: Sex reassignment
SRS: Sex reassignment surgery
GRS: Gender reassignment surgery
GAS: Gender assignment surgery

## Footnotes

1 We acknowledge that the use of various terms in this context, such as gender-affirming medical *treatment* versus gender-affirming medical *care*, can have different implications. Throughout this article, we use the term “gender-affirming medical treatment” (GAMT), to remain consistent with the current terminology used by WPATH and throughout the SOC8 [35, 96]. While an examination of the distinctions and nuances in the utilization of GAMC and GAMT would be worthwhile, such an analysis extends beyond the scope of this article.

2 In this article, we use TGD as an umbrella term referring to various forms of gender identities, roles, and expressions of and relations to gender that are different from those normatively expected of one’s assigned sex at birth.

3 The eligibility criteria for GAMT interventions have been widely critiqued by various scholars (e.g., [45, 97, 98]) for gatekeeping GAMT for TGD individuals. Critics argue that these criteria impose unnecessary barriers, restrict access to essential medical treatments, and undermine the self-determination of TGD individuals by subjecting them to extensive psychological evaluations.

4 The SOC8 was developed by a committee of 119 experts from a variety of disciplines and with diverse backgrounds. In order to determine the final version of the SOC8, the committee employed a Delphi technique in combination with existing scientific evidence [35].

5 Some articles employ outdated, inappropriate language to refer to TGD individuals and elements of GAMT. While we made efforts to exclude harmful language whenever possible, we maintain the original terminology when quoting these sources.

6 We recognize how drawing a distinction between these terms can be problematized [73, 76, 77] and acknowledge that using ‘trans’ and ‘queer’ as standalone categories undermines their intended purpose.

7 In the diagnosis of gender incongruence [44], the defining element of distress in order to access GAMT is removed, instead emphasizing the incongruence rather than the potential distress it may cause. In line with this, the SOC8 now recommends using the diagnosis of gender incongruence to facilitate access to GAMT [35]. However, in practice, gender dysphoria, accompanied by a narrative of distress, remains prevalent in clinical settings and continues to influence eligibility for GAMT.

8 It is important to note that some of the studies referenced in the “Research Section” in the SOC8 Adolescents chapter were conducted at a time when concepts such as gender fluidity and non-binariness were not recognized or considered in research, especially in Cohen-Kettenis and Van Goozen [64] and Smith et al. [29, 68]. As a result, the utilized scales and discussed outcomes predominantly reflect a binary understanding of gender.

9 Most authors do not mention race. Some mention it as a demographic [20, 66, 67, 72, 73, 75]. Only Tordoff et al. [72] discuss considering it as a covariate, while none of the studies discuss it in depth. However, race and gender are deeply intertwined and influence societal expectations and perceptions of gender.

10 See also [9, 99].
